# Cochlear Dysfunction in Children following Cardiac Bypass Surgery

**DOI:** 10.5402/2012/375038

**Published:** 2012-07-01

**Authors:** Mona M. El Ganzoury, Terez B. Kamel, Lobna H. Khalil, A. M. Seliem

**Affiliations:** ^1^Department of Pediatrics, Ain Shams University, Cairo, Egypt; ^2^Department of Audiology, Ain Shams University, Cairo, Egypt

## Abstract

*Background*. Sensorineural hearing loss after procedures including extracorporeal circulation and hypothermia is greater than general population. Mild hypothermia has a protective role on cochlea; however, deep hypothermia may result in cochlear injury. This research aimed at assessing auditory function in children after open heart surgery in relation to different hypothermic techniques. *Subjects and Methods*. Forty children with acyanotic heart diseases who underwent open heart surgery were included: group I: twenty patients subjected to mild hypothermia (33° to 37°C), group II: twenty patients subjected to moderate hypothermia (28° to 32°C). Audiological assessment included basic evaluation and otoacoustic emissions. *Results*. Both groups had distortion-product otoacoustic emissions (DPOAEs) amplitude >3 dB SPL at all frequencies. However, group II showed lower amplitude at overall and at high frequencies (4.416–8.837 KHz) than group I. Transient evoked otoacoustic emissions (TEOAEs) showed partial pass in three patients of group I (15%) and in 15 patients of group II (75%). Moreover, group II showed statistical significant reduction in overall TEOAEs amplitude as well as at high frequencies (2–4 KHz). *Conclusions*. Patients exposed to moderate hypothermic technique had subtle cochlear dysfunction. Otoacoustic emissions should be used for early detection of subtle cochlear dysfunction in operated cardiac children.

## 1. Introduction

The risk of sensorineural hearing loss after surgical procedures including extracorporeal circulation and hypothermia is estimated at 0.14% in the literature, a rate six times greater than the risk in general population [1]. In cardiopulmonary bypass most patients are subjected to one of two techniques regarding body temperature either mild (normothermic) technique in which body temperature ranges from 32° to 37°C or moderate hypothermic technique in which body temperature ranges from 28° to 32°C [2]. Few studies revealed that hypothermia has a protective role on the cochlea and could prevent its damage during long-lasting operations performed in extracorporeal circulation [3]. However, extracorporeal circulation with deep hypothermia may result in cochlear cells injury [4].

Otoacoustic emissions (OAEs), described by Kemp in 1978 [5], arise apparently in the outer hair cells (OHCs) and possibly represent the rapid contractions of this cell group. They are acoustic phenomena of cochlear nature, which reverberate through the ossicles in the middle ear and are transmitted to the ear canal, where they can be captured through a microphone [5]. They are objective, noninvasive, and rapid measures used to determine cochlear outer hair cell function [6].

Transient evoked otoacoustic emissions (TEOAEs) are frequency dispersive responses following a brief acoustic stimulus such as click or tone burst [7]. Distortion-product emissions (DPOAEs) are defined as acoustic energy in the ear canal arising from the nonlinear interaction of two simultaneously applied pure tones within the cochlea. Otoacoustic emissions (OAEs) testing can identify the cochlear component of a hearing disorder and monitor objectively minute changes in cochlear status undetectable by other audiological methods [5, 7, 8].

 Could DPOAEs and TEOAEs be utilized to monitor minute cochlear changes in children in such risk after open heart surgery? It has been established in the literature that hypothermia impacts cochlear potential, spontaneous OAE, and evoked transients, both in animal and human models during cardiopulmonary bypass surgeries [9–11]. However, there are no reports on altered TEOAEs or DPOAEs results after hypothermia in humans. So this research aimed to assess the effect of different hypothermic techniques on cochlear functions in children after open heart surgery using otoacoustic emissions.

## 2. Subjects and Methods

During the period from May, 2008 to November, 2010, this cross-sectional study was carried out as a cooperation between the Pediatric Cardiology Clinic, Children's hospital and the Audiology Unit, Faculty of Medicine, Ain Shams University, Cairo, Egypt.

 Forty children with various acyanotic heart diseases who underwent open heart surgery were enrolled in this study on the fifth to seventh day postoperatively. Their pre- and postoperative saturation of oxygen (SaO_2_) by pulse oximetry ranged from 92 mmHg to 98 mmHg. Hypoxemia was considered for SaO_2_ values less than 85 mmHg [12] to be excluded from the study.

During surgery, all studied patients were premeditated with rectal administration of flunitrazepam (0.5 mg/kg) one hour before surgery. Induction of anesthesia was achieved by intravenous administration of thiopental (5–7 mg/kg) and fentanyl (15–30 pg/kg). Achievement of neuromuscular blockade was done by pancuronium (0.15 mg/kg) then followed by endotracheal intubation. Anesthesia was maintained by intermittent administration of intravenous fentanyl, sometimes supplemented by a mixture of nitrous oxide and oxygen isoflurane. Esophageal temperature was used as a measure of blood perfusate temperature. Rectal and tympanic temperatures were also monitored to evaluate depth of cooling. One of the third-generation cephalosporin antibiotics was given.

According to degree of hypothermia, they were subdivided in two groups: normothermic group I: twenty patients who were subjected to mild hypothermic technique (33° to 37°C) and hypothermic group II: twenty children who were subjected to moderate hypothermic technique (28° to 32°C). 

An informed written consent was obtained from all studied children's parents. The study was ethically approved by the Pediatric Department Board. 

### 2.1. Audiological Assessment 

Patients had history of recurrent otitis media or family history of deafness, and those showed abnormal tympanometry were excluded from the study. Otoscopic examination, pure tone, and speech audiometry using Madsen Electronic audiometer, model Orbiter 922, in a sound-treated room IAC model 1602 were done. The pure tone thresholds were detected for each octave frequency between 0.25 and 8.00 kHz. Immittancemetry including tympanometry and acoustic reflex threshold testing using the Grason Stadler impedance meter model GSI 33 was performed. 

### 2.2. TEOAEs Recording Sand Interpretations 

Transient evoked otoacoustic emissions (TEOAEs) were obtained by using Smart Intelligent OAEs analyzer, version 3.02. Subjects were resting in a sound-treated room. A probe fitted to the tested ear delivered acoustic stimuli at an average of 80 dB SPL, and responses (echo levels) were recorded at 5 frequency bands over a range of 1 to 4.0 kHz responses. 

The results of TEOAEs were interpreted according to Maxon et al. [13] into one of three categories: pass; response was 3 dB or above signal-to-noise ratio (SNR ≥ 3 dB) in all test frequency bands, partial pass; response was present in at least one of the test frequency bands but not in all frequency bands, and fail; no response is present in any of the test frequency bands.

### 2.3. DPOAEs Testing

Two primary frequencies, f1 and f2, were presented simultaneously with f2/f1 equaling 1.22. Eight points per octave were measured and plotted as function of f2 ranging from 0.5 to 8.0 kHz. Primary tone level combinations L1/L2 = 75/65 dB SPL. DPOAEs measurements at 2f1/f2 were considered present when the emission amplitude at all individual frequencies was at least 3 dB higher than its associated noise amplitude (SNR ≥ 3 dB).

### 2.4. Statistical Analysis 

We analyzed the data using an IBM computer SPSS (Statistical Program for Social Science) version 15; Chicago, IL, USA, as follows: quantitative variables as range and mean (SD), qualitative variables as numbers and percentage were calculated. Student's *t*-test was used for two independent means with normal distribution while comparison between two independent groups for non-parametric data was by using the wilcoxon rank sum test. *P* < 0.05 was considered a significant.

## 3. Results

Forty children with various acyanotic heart diseases (Table 1) who underwent open heart surgery were enrolled in this study. The cardiopulmonary bypass surgery with extracorporeal circulation lasted no more than two hours. Their age ranged from 3 to 12 years; they were 23 males (57.5%) and 17 females (42.5%). Twenty-one patients (70%) had weight below 5th centile, and 17 patients (56.7%) had height below 5th centile.

These children were subdivided in two groups according to degree of cooling: normothermic group I: twenty patients who were subjected to mild hypothermic technique (33° to 37°C), they were 11 males and 9 females, and their mean age (SD) was 7.55 (4.41) years and hypothermic group II: twenty children who were subjected to moderate hypothermic technique (28° to 32°C); they were 12 males and 8 females with a mean age (SD) 7.75 (4.41) years.

**Figure 1:**
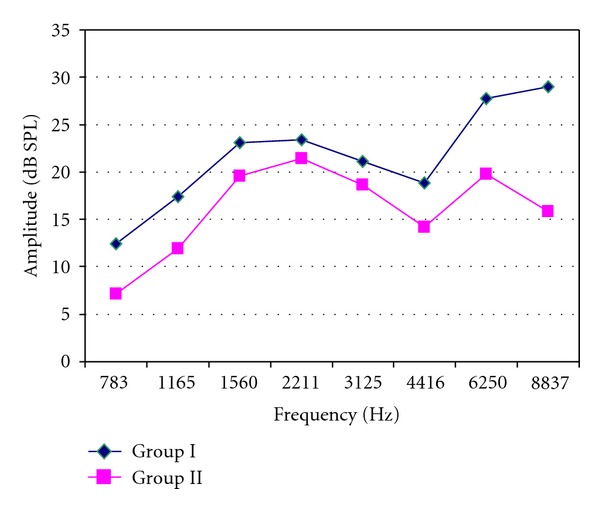
DPOAES amplitude levels at different frequencies. group I: normothermic; group II: hypothermic; dB: decibel; SPL: sound pressure level.

All children had normal hearing sensitivity as revealed by pure tone and speech audiometry with no statistical difference between the two groups (Table 2). All children had type (A) tympanograms and normal acoustic reflex thresholds indicating normal middle ear functions. All had DPOAEs amplitude >3 dB at all frequencies. However, group II (hypothermic group) showed a statistically significant reduction of amplitude levels at high frequencies (4.416–8.837 kHz; *P* < 0.01; Table 3, Figure 1).

**Figure 2:**
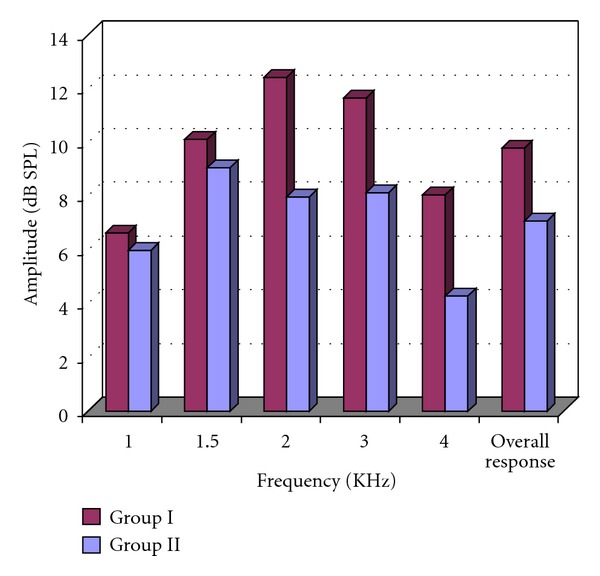
TEOAES amplitude levels at different frequencies and overall response level.group I: normothermic; group II: hypothermic; dB: decibel; SPL: sound pressure level.

TEOAEs results showed partial pass in only three patients (15%) of the normothermic group I and in 15 patients (75%) of the hypothermic group II. Moreover, the hypothermic group II showed a statistical significant reduction in the overall amplitude as well as at high frequencies “2–4” kHz (*P* < 0.01, Table 4, Figure 2). 

## 4. Discussion

Among more than 40,000 infants born with congenital heart disease annually in United States, one-third requires surgery during the first year of life [14]. Surgical techniques have reduced rates of mortality, so, attention has shifted toward neurodevelopmental morbidity among the growing survivors. Long-term deficits in cognition, attention, and neuromotor functioning likely reflect the cumulative impact of features present before, during, and after surgical intervention [14].

Cardiopulmonary bypass management in infants and children often requires the use of hypothermia with occasional periods of circulatory arrest and represents marked physiologic extremes of temperature and perfusion [15]. Moreover, risk of sensorineural hearing loss after such surgical procedures is estimated at a rate six times greater than general population [1].

The eligibility of this cross-sectional study was to assess the behavior of the OHCs after heart surgery where mild to moderate hypothermia and extracorporeal circulations are used. This may help for early detection of subtle cochlear dysfunction in those operated cardiac children. It included 40 patients with acyanotic heart disease, of them 23 (57.5%) were males and 17 (42.5%) were females. This agreed with Marelli et al. [16], who concluded that more male children undergo congenital heart disease surgery (54.7%). Moreover, similar to several studies [17–19], our study reported ventricular septal defect (VSD) as the commonest lesion (70%).

In concordance with many studies [19, 20], patients' group in current study showed growth failure. Infants with congenital heart disease (CHD) are prone to malnutrition for several reasons including decreased energy intake, increased energy requirements, or both. Severity of malnutrition ranges from mild undernutrition to failure to thrive. This can have a notable effect on the outcome of surgery, increasing morbidity and mortality [21]. 

In the current work all patients had postoperatively normal hearing sensitivity. Sudden deafness is uncommon complication following cardiac surgery with extracorporeal circulation and was first reported by Arenberg et al. in 1972 [22]. In 1980, Plasse et al. [23] reported seven cases of unilateral sensorineural hearing loss (SNHL) from a total of 7000 patients, immediately after cardiac operations for coronary artery bypass or congenital heart disease. 

However, in agreement with the present study, Brownson et al. [24] performed audiometry in 50 postoperative patients and found no changes in hearing while Shapiro et al. [25] reported two patients with unilateral severe SNHL immediately after cardiac operations, but a controlled follow-up study revealed modest bilateral changes in thresholds. These findings probably represented perfusion problems.

In our work, although all the children had normal hearing sensitivity, DPOAEs of the hypothermic group II showed a significant statistically reduction of amplitude at high frequencies (4.416–8.837 kHz). These findings were confirmed by TEOAEs results that showed 75% partial pass response of the hypothermic group II, compared to 15% in normothermic group I. Moreover, the hypothermic group II had a significant reduction in the overall amplitude of TEOAEs as well as at high frequencies “2–4” kHz. 

Although TEOAEs have the advantage of evaluating middle-frequency (1–4 kHz), DPOAEs precisely detect cochlear dysfunction in a frequency-specific manner and are superior in measuring higher frequency ranges. OAEs' significant findings among the moderate hypothermic group II might imply impairment in the activation of cochlear OHCs generating OAEs. This impairment was evident mainly at the high frequency regions of the cochlea which may reflect more affection of the basal turn of the cochlea other than the rest of the cochlea. Such cochlear affection may be termed cochleotopic gradient of susceptibility [26].

Few researchers have studied the effects of temperature changes on cochlear function in humans using TEOAEs. Both Veuillet et al. [4] and Seifert et al. [11] reported TEOAEs alterations among the frequency components with cooling. Namysłowski et al. [3] assessed TEOAEs before and following surgery; TEOAEs showed no tendency to decrease in patients whose extracorporeal normothermia circulation time was between 1 and 2 hours. 

More recently, Borin and Cruz [27], using DPOAEs, reported that moderate hypothermia (28°-29°C) during heart surgery with extracorporeal circulation has led to a decrease in the amplitude of distortion product otoacoustic emissions, but they attributed that patients had increased risk of cochlear damage due to advanced age and associated diseases (high blood pressure, diabetes, and atherosclerosis).

When looking at papers on hearing loss after open heart surgery [22, 25], similar pathophysiological mechanisms associated with CNS injury can be found, the main being inner ear ischemia and hypoperfusion. Others such as Ness et al. [1] attributed cochlear injury after cardiopulmonary bypass more as a consequence of ototoxic drug use than the surgical procedure itself. Meri et al. [28] related cochlear injury to complement activation during cardiopulmonary bypass in children, another mechanism that may occur at a cochlear level. 

Although cochlear injury consequent to cardiopulmonary bypass has not been clearly defined, experimental animal studies have shown ischemic induced injury after reperfusion of the inner ear [9, 29]. Glutamate, an excitatory neurotransmitter in the cochlea, is thought to play an important role in the pathogenesis of ischemia-induced cochlear damage [30].

However, the role of mild hypothermia (33° to 37°C) in prevention of inner ear damage has been proved by many researchers [3, 4, 11, 27]. Moreover, Watanabe et al. [29] demonstrated that hearing loss and inner ear damage were completely prevented by preischemic mild hypothermia, while Hyodo et al. [30] considered that such protective effects were primarily through reduction of glutamate efflux. In addition, the protective role of postischemic mild hypothermia has been suggested through the attenuation of oxidative stress [31].

 Till now, evaluation of such protective role using moderate hypothermia (28° to 33°C) has not yet been investigated. So, such subtle cochlear dysfunction found in hypothermic group II might be explained by mild ischemia and/or hypoperfusion injury of the inner ear, which might have occurred during cardiopulmonary bypass surgery or related to the degree of cooling itself. Yet, the overall normal hearing sensitivity in both groups would reflect the protective value of using hypothermia during cardiopulmonary bypass surgery. 

In conclusion, patients exposed to moderate hypothermic technique during cardiac surgery had subtle cochlear dysfunction. Care should be taken for choice of moderate hypothermic technique in open heart surgeries for children at risk for sensorineural hearing loss. Otoacoustic emissions testing can be used routinely for early detection of subtle cochlear dysfunction in those operated cardiac children.

## Figures and Tables

**Table 1:** Frequency of cardiac lesions among patients' group (*n* = 40).

Group	Diagnosis	Number	%
Mild hypothermic (*n* = 20)	VSD	7	35.0
	ASD	5	25.0
	AS	5	25.0
	MR	1	5.0
	MS	2	10.0

Moderate hypothermic (*n* = 20)	VSD	7	35.0
	ASD	7	35.0
	AS	2	10.0
	MS	1	5.0
	MR	2	10.0
	MR + MS	1	5.0

VSD: ventricular septal defect, ASD: atrial septal defect, AS: aortic stenosis, MR: mitral regurg, MS: mitral stenosis.

**Table 2:** Pure tone audiometry and speech audiometry in both groups.^∗^

Frequency	Normothermic group I (*n* = 20)	Hypothermic group II (*n* = 20)
	mean (SD) (dB)	mean (SD) (dB)
250 Hz	9.7 (3.3)	11 (3.4)
500 Hz	10.7 (3.7)	11.2 (4.5)
1 kHz	9.7 (4.4)	12 (4.7)
2 kHz	11.2 (5.3)	9.7 (3.4)
4 kHz	10.2 (3.4)	10.7 (4.6)
8 kHz	12 (4.8)	11.2 (4.5)
SRT	9.5 (4.2)	9.5 (3.5)
WDS	98.8 (2.2)	98.4 (2.7)

^∗^ *P* > 0.05: nonsignificant, SRT: speech reception threshold, WDS: word discrimination scores, dB: decibel.

**Table 3:** DPOAES response levels at different frequencies in both groups.

Frequency (kHz)	Normothermic group I DPOAES	Hypothermic group II DPOAES	*P* value
	mean (SD) (dB SPL)	mean (SD) (dB SPL)	
7.83	12.38 (6.94)	7.10 (8.39)	0.01^∗^
1.165	17.35 (9.169)	11.95 (8.80)	0.01^∗^
1.560	23.08 (9.65)	19.58 (8.76)	0.09
2.211	23.40 (9.78)	21.45 (9.592)	0.37
3.125	21.10 (9.78)	18.68 (9.00)	0.25
4.416	18.85 (6.225)	14.20 (8.30)	0.00^∗^
6.250	27.75 (9.89)	19.75 (9.85)	0.00^∗^
8.837	28.95 (12.02)	15.85 (10.32)	0.00^∗^

^∗^Statistically significant; dB: decibel; SPL: sound pressure level.

**Table 4:** TEOAES response levels at different frequencies in both groups.

Frequency (kHz)	Normothermic group I TEOAES	Hypothermic group II TEOAES	*P* value
	mean (SD) (dB SPL)	mean (SD) (dB SPL)	
1	6.62 (5.79)	5.98 (5.61)	0.62
1.5	10.11 (5.79)	9.06 (6.56)	0.44
3	12.40 (12.40)	7.93 (7.93)	0.00^∗^
4	11.63 (5.54)	8.12 (5.64)	0.00^∗^
Overall response	8.04 (5.171)	4.28 (4.98)	0.00^∗^

^∗^Statistically significant; dB: decibel; SPL: sound pressure level.
